# Outer membrane vesicles of *Glaesserlla parasuis* activate the endosomal cGAS–STING–IRF3 pathway through nucleic acid payload delivery: a biological perspective on host defense protocol optimization

**DOI:** 10.1186/s13567-025-01553-5

**Published:** 2025-07-01

**Authors:** Kunli Zhang, Zeyi Sun, Xintong Kang, Keda Shi, Pinpin Chu, Dongxia Yang, Zhibiao Bian, Yan Li, Hongchao Gou, Zhiyong Jiang, Nanling Yang, Xia Zhou, Sutian Wang, Zhanyong Wei, Shaolun Zhai, Huahua Kang, Chunling Li

**Affiliations:** 1 Key Laboratory of Livestock Disease Prevention of Guangdong Province, Guangdong Provincial Observation and Research Station for Animal Disease, Institute of Animal Health, Guangdong Academy of Agricultural Sciences, Guangzhou, 510640 China; 2 State Key Laboratory of Livestock and Poultry Breeding, Guangdong Key Laboratory of Animal Breeding and Nutrition, Institute of Animal Science, Guangdong Academy of Agricultural Sciences, Guangzhou, 510640 China; 3 The College of Animal Science and Veterinary Medicine, Henan Agricultural University, Zhengzhou, 450046 China

**Keywords:** *Glaesserlla parasuis*, outer membrane vesicles, type I interferons, cGAS–STING

## Abstract

*Glaesserlla parasuis* (*G. parasuis*), a Gram-negative pathogen responsible for Glässer's disease, employs outer membrane vesicles (OMVs) as sophisticated nanoscale effectors to modulate host‒pathogen interplay. While bacterial OMVs are recognized as critical mediators of virulence dissemination, their functional orchestration in *G. parasuis* immunopathogenesis remains unclear. To date, few reports have focused on the relationships among *G. parasuis*, OMVs and host-susceptible cells; thus, more evidence is urgently needed to explore their crosstalk further. This study revealed a novel immune activation paradigm: both *G. parasuis* and its OMVs trigger robust type I interferon (IFN) responses via a DNA-sensing cascade. *G. parasuis* OMVs-Dio were internalized by macrophages in a time-dependent manner, partially via clathrin-mediated endocytosis but mainly via dynamin-dependent endocytosis. Studies have shown that IFNs play key antiviral roles in viral infections and important roles in bacterial infections. Our results suggested that IFNs inhibited *G. parasuis* adhesion and invasion of pulmonary alveolar macrophage (PAM) cells. Furthermore, by assessing the major components of OMVs, we confirmed that the DNA of *G. parasuis*, which is carried by OMVs, is the key component that induces the production of IFN in macrophages. The cGAS–STING–IRF3 pathway links the host’s recognition of *G. parasuis* OMVs to IFN production. Taken together, our data reveal that *G. parasuis* OMVs activate cGAS/STING/IRF3 signaling and induce IFN production, which then affects the adhesion and invasion of *G. parasuis*. The discovery of this vesicle-mediated nucleic acid delivery system redefines the pathogenesis framework for *G. parasuis* and provides a trans-species conceptual advance in understanding how Gram-negative pathogens exploit vesicular trafficking to manipulate host immunity.

## Introduction

Innate immunity is the front-line defense against viral and bacterial infections. Almost all nucleated cells in the body produce type I interferons (IFNs) in response to infection. IFNs play important roles in virus clearance, antigen presentation, the inhibition of cancer cell proliferation, and the positive feedback loop of type I IFN production. Although IFNs are known to have broad-spectrum antiviral activity, the role of IFNs in bacterial infection is still unclear. Unlike those of viral infection, the regulatory effects of type I IFNs on bacterial infections differ greatly among different species, replication processes, virulence factors, infection routes and infection sites. Type I IFNs can promote the immune escape of bacteria and accelerate bacterial infection and spread. Infections by intracellular or extracellular bacteria such as *Listeria monocytogenes*, *Francisella tularensis*, *Salmonella enterica* serovar *Typhimurium*, and *Staphylococcus aureus* can be exacerbated by IFNs [1–3]. However, in other bacterial infections, type I interferons can effectively inhibit bacterial proliferation and control the course of the disease, such as *Legionella pneumophilia*, *Streptococcus pyogenes*, and *Helicobacter pylori* infections [4, 5]. The effect of interferon on other bacterial pathogens, including bacteria of swine origin, is still unknown.

*Glaesserlla parasuis* is a common opportunistic pathogen in the upper respiratory tract of healthy swine [1]. It is a Gram-negative short bacilli with variable morphology. When pigs are stressed, immune compromised, or otherwise infected, *G. parasuis* causes Glässer’s disease, which is characterized by elevated body temperature, joint swelling, breathing difficulties, fibrinous polyserositis, and even meningitis. The incidence rate of this disease is generally between 10 and 15%, and in severe cases, it can reach 80%. The mortality rate can exceed 50%. It has been the “number one killer” among bacterial diseases in the pig industry and has severely restricted the development and expansion of large-scale pig farming in China. Outer membrane vesicles (OMVs) are vesicular nanoparticles produced by Gram-negative bacteria. Our previous studies revealed that the *G. parasuis* H45 strain can secrete OMVs with sizes ranging from approximately 20 to 160 nm [6]. OMVs have been reported to play crucial roles in host‒microbe interactions. During *Helicobacter pylori* infection, OMVs transport surface peptidoglycans to the cytoplasm through lipid raft-mediated endocytosis and activate the NF-κB signaling pathway via NOD1, leading to inflammatory cytokine production [7]. OMVs secreted by *Escherichia coli* mediate the transfer of LPS from the extracellular space to the cytoplasm, activate the NLRP3 inflammasome, and cause host cell injury and inflammation [8]. Owing to the special structure of OMVs, in addition to delivering virulence factors to host cells such as the other secretory systems of bacteria, they can also act as carriers of pathogenic bacterial nucleic acids that can regulate the natural immune responses of host cells. Reports have indicated that *actinomycetemcomitans* (Aa) OMVs transfer extracellular RNAs (exRNAs) to activate the TLR8 and NF-κB signaling pathways in macrophages [9] and that *Pseudomonas aeruginosa* OMVs carry sRNAs to attenuate keratinocyte-derived chemokine cytokine secretion and neutrophil infiltration in mouse lungs [10]. However, the mechanism by which *G. parasuis* OMVs induce host innate immunity remains unclear.

In this study, we first reported that, consistent with *G. parasuis* infection, OMVs could also induce IFN production in macrophages. Interestingly, the DNA of *G. parasuis* carried by OMVs is the key component by which *G. parasuis* induces macrophages to produce type I interferon by activating the cGAS–STING–IRF3 pathway. Furthermore, bacterial adherence and invasion assays revealed that type I IFNs inhibited *G. parasuis* adhesion to and invasion of PAM cells. Overall, this is the first report showing that OMVs act as carriers to deliver *G. parasuis* DNA and induce macrophages to produce type I interferons and that type I IFNs inhibit *G. parasuis* infection. The discovery of this vesicle-mediated nucleic acid delivery system redefines the pathogenesis framework for *G. parasuis* and provides a trans-species conceptual advance in understanding how Gram-negative pathogens exploit vesicular trafficking to manipulate host immunity.

## Materials and methods

### Bacterial strain and growth conditions

The *Glaesserlla parasuis* H45 strains (serovars 5) were provided by the Institute of Animal Health of the Guangdong Academy of Agricultural Sciences. The bacteria were cultured in TSB supplemented with 10% bovine serum and 1 mg/mL nicotinamide adenine dinucleotide (NAD). *G. parasuis* were cultured overnight at 37 °C with 200 rpm circular agitation and harvested when the optical density at 600 nm (OD_600_) reached 0.7–0.9. The *G. parasuis* were stored at −80 °C with 20% glycerol.

### Cell lines

3D4/21 cells and RAW264.7 cells were purchased from the American Type Culture Collection (ATCC). Pulmonary alveolar macrophages (PAMs) were isolated from 4-week-old piglets [11]. 3D4/21 cells and RAW264.7 cells were cultured in Dulbecco’s modified Eagle’s medium (DMEM), and PAMs were cultured in RPMI-1640 medium. All the cultured cells were supplemented with 10% fetal bovine serum (FBS), 100 U/mL penicillin, and 100 μg/mL streptomycin, and the cells were cultured at 37 °C under 5% CO_2_. The animals used in this research were approved by the Animal Welfare and Ethics Committee of the Animal Health Institute of Guangdong Academy of Agricultural Sciences with approval number YC-PT2020050.

### Reagents and antibodies

cGAMP (SML1232) was purchased from Sigma‒Aldrich (St. Louis, MO, USA). Anti-IRF3 (D83B9, 4302), anti-IRF3 phosphorylated at S396 (4D4G, 4947), anti-STING (D2P2F, 13647), anti-TBK1 (D1B4, 3504), and anti-TBK1 phosphorylated at S172 (D52C2, 5483) were purchased from Cell Signaling Technology (Danvers, MA, USA). Anti-actin (81115-1-RR) was purchased from Proteintech Group (Rosemont, IL, USA). Horseradish peroxidase (HRP)-conjugated anti-mouse (A4416), HRP-conjugated anti-rabbit (A0545) and Alexa Fluor 488-labeled goat anti-rabbit IgG were purchased from Thermo Fisher Scientific (Waltham, MA, USA).

### Purification of OMVs

OMVs were collected from the supernatant of *G. parasuis H45 strain* cultures as previously described [6]. Briefly, *G. parasuis* was inoculated in 1000 mL of TSB broth at 37 °C with shaking until the OD_600_ reached 1.0, after which the bacterial supernatants were collected and concentrated with a 100 kDa hollow-fiber membrane. The remaining bacteria in the concentrated supernatants were also removed with 0.22 μm membranes. The coarse OMVs were obtained via ultracentrifugation (150 000 × *g* for 4.5 h at 4 °C). The coarse OMVs were resuspended in sterile phosphate-buffered saline (PBS) and purified via density gradient centrifugation with OptiPrep™ from 45 to 20% (v/v) [36]. The OMV-containing fractions were collected and diluted with sterile PBS. Then, ultracentrifugation (200 500 × *g* × 2 h, 4 °C) was performed to remove the OptiPrep™ solution. After the sterility of the purified OMVs was checked, they were resuspended in sterile PBS and stored at −80 °C.

DNA-free OMV preparation. The DNA-free OMVs were prepared as previously described [9]. The detailed process used the following steps. First, 500 μg of OMVs was digested with 2 μL of DNase (5 U/μL, Takara) at 37 °C for 2 h to remove surface DNA. Second, the OMV samples were ultrasonically cleaved and then treated with 2 μL of DNase to remove DNA from the cavity. Third, the OMVs were heated to 85 °C for 15 min to inactivate the DNase.

### Quantification of the DNA of OMVs

A PicoGreen assay kit (Invitrogen, USA) was used to quantify the DNA of the OMVs. The methods used were previously described [12]. The sample was pretreated according to the following steps. (1) The purified OMVs (30 μg) were centrifuged at 150 000 × *g* for 30 min at 4 °C and divided into three groups. (2) The three groups were resuspended in 1 mL of 3 different solutions (50 mM HEPES–150 mM NaCl solution; GES lysis reagent; DNA enzyme reagent, which contains 10 μg/mL DNase I). (3) The samples were incubated on a shaker at 37 °C for 30 min. Finally, the samples were centrifuged at 150 000 × *g* for 30 min at 4 °C, and the supernatants and precipitates of each group were collected separately for DNA quantification. Finally, the results of different samples were measured with a microplate reader.

### Bacterial genome extraction, RNA extraction and qPCR

The genomic DNA of *G. parasuis* (H45) was extracted via the Bacterial Genome Extraction Kit (Takara, Japan). A Total RNA Kit I (Omega) was used to extract total RNA from the cells, and a PrimeScript™ RT Reagent Kit (Takara) was used to reverse transcribe the RNA. The reverse transcription products were amplified via a LightCycler® 480 Instrument II qPCR system with SYBR® Premix Ex Taq™ II (Takara). The above operations were carried out according to the manufacturer’s instructions. The data were normalized to the level of PPIA gene expression in each sample. The qPCR sequences of primers used were as follows: swine-IFNβ-F: 5′-GTGGAACTTGATGGGCAGAT-3′, swine-IFNβ-R: 5′-TTCCTCCTCCTCCATGATTTCCTC-3′; swine-cGAS-F: 5′-GGAGCCCTGCAGTAACACTT-3′, swine-cGAS-R: 5′-GCTCCAAGCCACTGACTGAT-3′; swine-PPIA-F: 5′-AAGGTTCCTGCTTTCACAGAATAAT-3′, swine-PPIA-R: 5′-AATTTCTCTCCATAGATGGACTTGC-3′; Mus-IFNβ-F: 5′-CCCTATGGAGATGACGGAGA-3′; and Mus-IFNβ-R: 5′-CTGTCTGCTGGTGGAGTTCA-3′.

### Confocal microscopy

RAW264.7 cells were grown on confocal dishes and separately divided into a mock group or stimulated groups. The stimulated groups were treated with *G. parasuis* at an MOI of 10, 25 μg/mL OMVs or 10 μg/mL cGAMP for 12 h. Then, the cells were fixed in 4% paraformaldehyde for 30 min and washed once with PBS. Second, the cells were permeabilized for 20 min in 0.3% Triton X-100 in PBS and blocked with 5% BSA in PBS for 1 h. Third, the cells were incubated with anti-STING antibodies for 1 h and stained with Alexa Fluor 488-labeled goat anti-rabbit IgG for 1 h. Finally, DAPI was used to stain the nuclear DNA. The subcellular localization of STING was visualized with a Zeiss LSM-710 laser scanning fluorescence microscope (Carl Zeiss AG, Oberkochen, Germany) under a 63× oil objective.

### Western blotting and ELISA

Western blot analysis was performed as previously described [13]. Briefly, equal amounts of cell lysates were resolved via 10‒12% sodium dodecyl sulfate‒polyacrylamide gel electrophoresis (SDS‒PAGE) and then transferred to a polyvinylidene difluoride (PVDF) membrane (Millipore). The membranes were blocked with 5% BSA in PBST for 1 h. After washing with PBST 3 times, the primary and secondary antibodies were used separately. The western blot results were visualized via a chemiluminescence imaging system (Fine-do X6; Tanon, China).

The concentrations of IFN-β (pbl) and IFN-α (Ray Bio) in the cell culture supernatants were measured via ELISA kits according to the manufacturer’s instructions.

### Adherence and invasion assays

The adherence and invasion assays were performed according to previous studies [14, 15]. The detailed method was as follows. PAM cells were seeded into 12-well tissue culture plates. Prior to infection, the cells were washed with sterile PBS to remove antibiotics and then inoculated with *G. parasuis* (MOI = 10). The cells were incubated for 2 and 4 h for adherence and invasion, respectively, after which the cell culture was removed, and the cells were washed five times with PBS to remove nonspecifically attached bacteria. For the invasion assay, the cells were treated with complete growth medium (including 100 U/mL penicillin G and 100 μg/mL gentamicin) for 1 h to kill any extracellular *G. parasuis* and then washed five times with PBS [14, 16–20]. The above cells were then incubated for 10 min at 37 °C with 100 μL of 0.25% trypsin. Following trypsin treatment, 800 μL H_2_O was added to disperse the cells. After the dispersed cells were diluted 10 times, 100 times, 1000 times, and 10 000 times, they were applied to the TSA plate. The colonies were counted after 24 h of incubation in a 37 °C incubator.

### Statistical analysis

Statistical analyses were performed via a Student’s *t test* or *one-way* or *two-way ANOVA* followed by *the Bonferroni post-test*. *P* values less than 0.05 were considered statistically significant. The numeric data were analyzed via GraphPad Prism software (USA).

## Results

### *Glaesserlla parasuis* induces IFN production in macrophages

**Figure 1:**
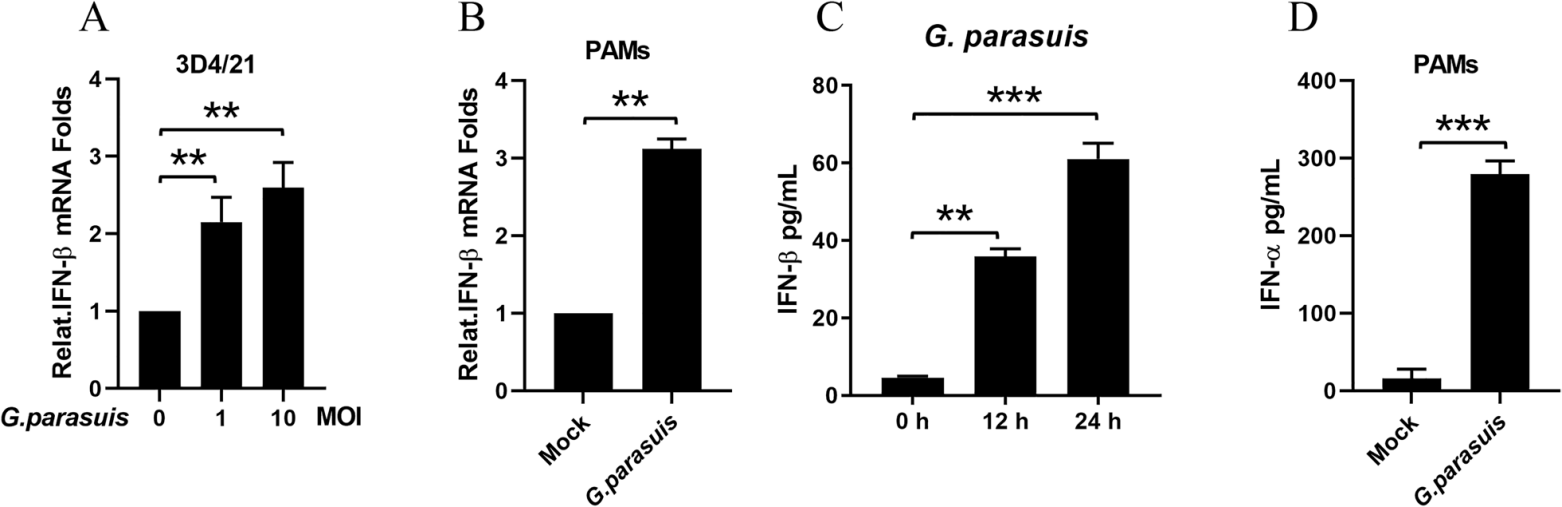
***G. parasuis* induces IFN production in macrophages. A** The 3D4/21 cells were infected with *G. parasuis* at MOIs of 0, 1 and 10 for 16 h. The mRNA levels of IFN-β in the *G. parasuis*-infected cells were detected via qPCR. **B** PAMs were either mock-infected or infected with *G. parasuis* at an MOI of 1 for 16 h. The mRNA levels of IFN-β in *G. parasuis*-infected cells were detected via qPCR. **C** RAW264.7 macrophages were either mock-infected or infected with *G. parasuis* at an MOI of 1 at different times (12 h or 24 h). The IFN-β levels in the cell supernatants were analyzed via ELISA. **D** PAMs were either mock-infected or infected with *G. parasuis* at an MOI of 1 for 24 h. The IFN-α levels in the cell supernatants were analyzed via ELISA. **p < 0.01, ***p < 0.001, one-way ANOVA followed by the Bonferroni post-test (**A** and **C**) or Student’s *t* test (**B** and **D**). The data are expressed as the means ± SDs of triplicate assays (**A**–**D**).

To determine whether *G. parasuis* (*G. parasuis*) induces IFN production, 3D4/21 cells were infected with *G. parasuis* at MOIs of 0, 1 or 10 for 16 h. Then, the cells were collected to determine the mRNA levels of IFN-β via qPCR. As shown in Figure 1A, G*. parasuis* infection induced IFN-β mRNA transcription in a dose-dependent manner. PAMs are the target cells of *G. parasuis* infection, so we used *G. parasuis*-infected PAMs at an MOI of 1. Infection with *G. parasuis* also increased the IFN-β mRNA transcription levels in PAMs (Figure 1B). In addition, *G. parasuis* were mock-infected or infected with RAW264.7 macrophages and PAMs for 24 h. The supernatants were collected, and the contents of IFN were determined via ELISA. The results in Figures 1C, D were consistent with the mRNA transcription results. Taken together, our data indicate that *G. parasuis* infection induces IFN production in macrophages.

### OMVs of *G. parasuis* induce IFN production in macrophages

**Figure 2:**
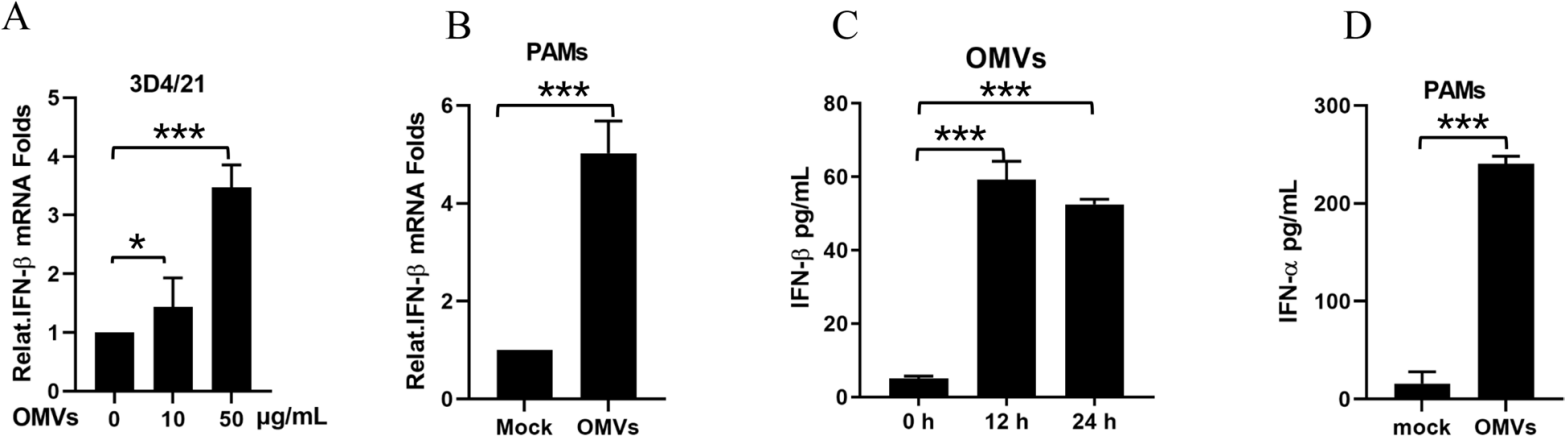
**OMVs of** ***G. parasuis* induce IFN production in macrophages. A** The 3D4/21 cells were treated with *G. parasuis* OMVs at 0, 10 and 50 μg/mL for 16 h. The mRNA levels of IFN-β in the OMV-treated cells were detected via qPCR. **B** PAMs were either mock-treated or treated with *G. parasuis* OMVs at 50 μg/mL for 16 h. The mRNA levels of IFN-β in the OMV-treated cells were detected via qPCR. **C** RAW264.7 macrophages were either mock-treated or treated with *G. parasuis* OMVs at 50 μg/mL for different durations (12 h or 24 h). The IFN-β levels in the cell supernatants were analyzed via ELISA. **D** PAMs were either mock-treated or treated with *G. parasuis* OMVs at 50 μg/mL for 24 h. The IFN-α levels in the cell supernatants were analyzed via ELISA. ***p < 0.001, one-way ANOVA followed by the Bonferroni post-test (**A** and **C**) or Student’s *t* test (**B** and **D**). The data are expressed as the means ± SDs of triplicate assays (**A**–**D**).

OMVs constitute a new independent secretory system of Gram-negative bacteria that play important roles in quorum sensing, the stress response, nutrient acquisition, adhesion and virulence factor transfer [21]. In a previous study, we confirmed that *G. parasuis* secretes OMVs during in vitro growth and that the size of *G. parasuis* OMVs ranges from approximately 50–100 nm in diameter [6]. As OMVs have a nanoclike structure, they can enter cells and activate various cellular immune responses. Moreover, we found that *G. parasuis* OMVs induce strong inflammatory responses in macrophages [6]. Therefore, we hypothesized that the OMVs of *G. parasuis* might also induce IFN production in macrophages. To test this hypothesis, we obtained OMVs from *G. parasuis* via density gradient centrifugation and collected the cells after 16 h of gradient infection. The qPCR results revealed that the OMVs of *G. parasuis* infection induced IFN-β mRNA transcription in a dose-dependent manner. The IFN-β mRNA transcription levels in PAMs were also detected after infection with 50 μg/mL OMVs for 16 h (Figure 2B). In the macrophages, including RAW264.7 cells and PAMs, the contents of IFN-β and IFN-α in the supernatant after OMV infection for 24 h were determined via ELISA (Figures 2C, D). The qPCR and ELISA results show that with *G. parasuis* infection, OMVs can induce IFN production in macrophages.

### The DNA of *G. parasuis* OMVs induce macrophages to produce IFNs

**Figure 3:**
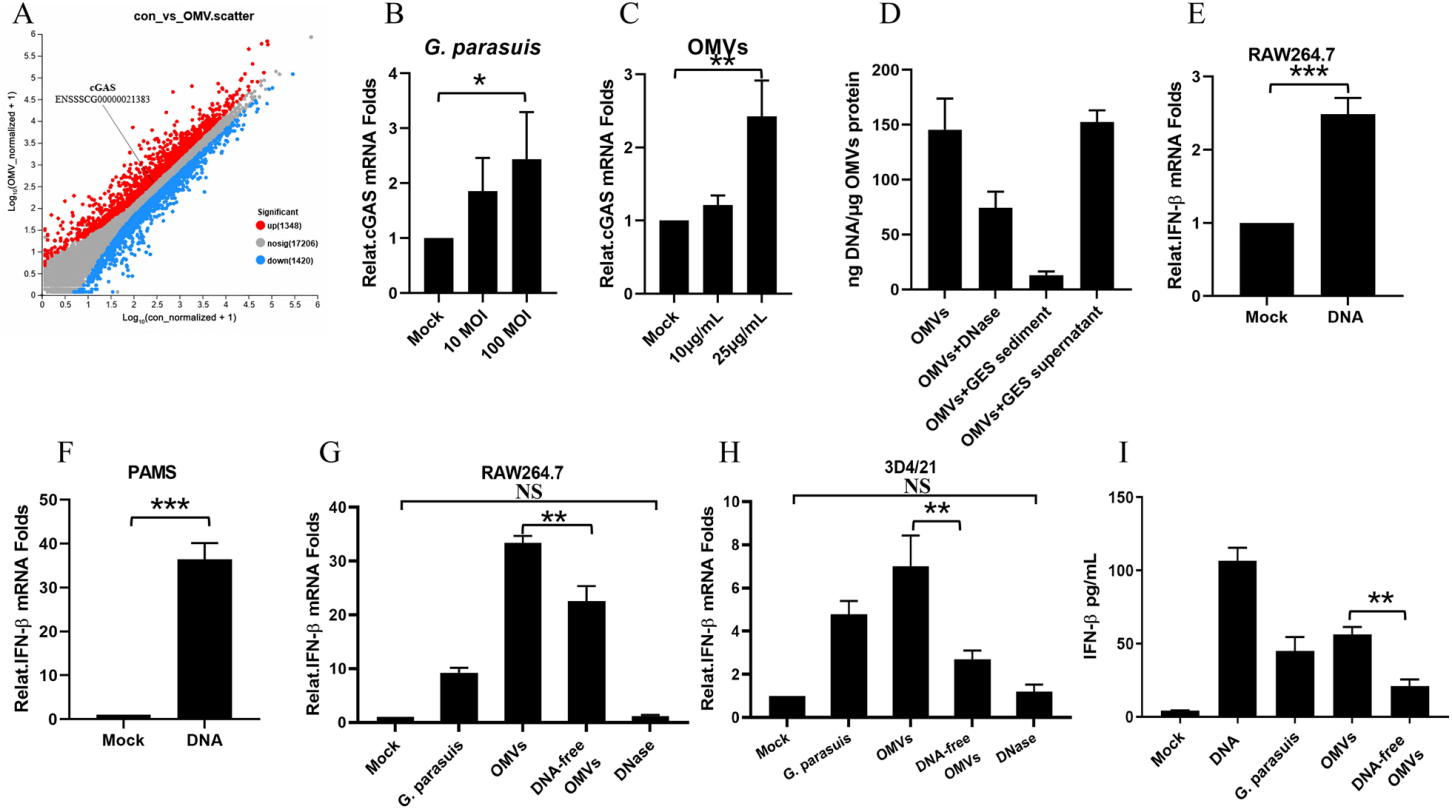
**DNA is the key component of** ***G. parasuis* OMVs that induces macrophages to produce type I interferon. A** PAMs were either mock-stimulated or stimulated with *G. parasuis* OMVs (50 μg/mL) for 12 h. The cells were collected, and the RNA-seq data were analyzed via majorbio. Scatter diaGram representing genes that are differentially regulated by stimulation with *G. parasuis* OMVs. The red dots represent upregulated genes, whereas the blue dots correspond to genes whose expression is differentially downregulated. The cGAS gene point is indicated by a black line. **B** PAMs were either mock-infected or infected with *G. parasuis* at MOIs of 0, 10 and 100 for 16 h. The mRNA levels of cGAS in *G. parasuis*-infected cells were detected via qPCR. **C** PAMs were either mock-stimulated or stimulated with OMVs at 0, 10 or 25 μg/mL for 16 h. The mRNA levels of cGAS in the OMV-stimulated cells were detected via qPCR. **D** The *G. parasuis* DNA contents on the OMV surface and inside the cavity were detected via ultraviolet spectrophotometry. **E** RAW264.7 cells were stimulated with 0 or 2 μg/mL *G. parasuis* DNA for 16 h. The mRNA levels of IFN-β in the cells were detected via qPCR. **F** PAMs were stimulated with 0 or 2 μg/mL *G. parasuis* DNA for 16 h. The mRNA levels of IFN-β in the cells were detected via qPCR. **G** RAW264.7 cells were stimulated with *G. parasuis* (MOI of 10), OMVs (50 μg/mL), or OMVs without nucleic acid (50 μg/mL) or inactivated with DNase (10 U) for 12 h. The mRNA levels of IFN-β in the cells were detected via qPCR. **H** 3D4/21 cells were treated with *G. parasuis* (MOI of 10), OMVs (50 μg/mL), or OMVs without nucleic acid (50 μg/mL) or inactivated with DNase (10 U) for 16 h. The mRNA levels of IFN-β in the cells were detected via qPCR. **I** RAW264.7 cells were stimulated with *G. parasuis* (MOI of 10), OMVs (50 μg/mL) or OMVs without nucleic acid (50 μg/mL) for 24 h. IFN-β levels in the cell supernatants were analyzed via ELISA. *p < 0.05, **p < 0.01, ***p < 0.001; one-way ANOVA followed by the Bonferroni post-test (**B**–**C**, **G**–**I**) and Student’s *t* test (**E** and **F**). The data are expressed as the means ± SDs of triplicate assays (**B**–**I**).

OMVs are composed of lipopolysaccharides (LPS), phospholipids, peptidoglycans, outer membrane proteins, cell wall components, nucleic acids (DNA, RNA) and ionic metabolites [22]. LPS, RNA and DNA are the main pathogen-associated molecular patterns (PAMPs) that can be recognized by pattern-recognition receptors (PRRs), such as Toll-like receptors (TLRs), retinoic acid-inducible gene I (RIG-I)-like receptors (RLRs) and DNA sensors, to induce IFN production. To assess which major components of OMVs induce type I interferon production, we performed transcriptomic analysis after the PAMs were infected with OMVs for 12 h. Compared with mock PAMs, OMVs significantly upregulated cGAS gene expression by 1.1275-fold in PAM cells (Figure 3A). The mRNA transcription level of cGAS was also detected after infection with *G. parasuis* or OMVs for 16 h. As shown in Figures 3B and C, the mRNA transcription level of cGAS increased with the content of *G. parasuis* and OMVs. Viral DNA, retroviral DNA, bacterial DNA, and even the host’s own DNA, which is located in the cytoplasm, can be recognized efficiently by cGAS. Once cGAS recognizes cytoplasmic DNA, STING is activated to induce type I IFNs and inflammatory cytokine production. We examined the contents of *G. parasuis* DNA carried on the OMV surface and inside the cavity via ultraviolet spectrophotometry. OMVs (1 μg/mL) contained approximately 135.2 ng of *G. parasuis* DNA, of which approximately 91% was wrapped in the vesicle cavity, and 9% of the DNA was located on the surface of the vesicle (Figure 3D). The DNA of *G. parasuis* was extracted and used to stimulate RAW264.7 cells and PAMs for 16 h. As expected, the DNA of *G. parasuis* induced type I interferon transcription in the macrophages (Figures 3E, F). To confirm whether the OMVs carry DNA that has the capacity to induce type I IFN production, the OMVs were digested with DNase at 37 °C for 2 h to remove the surface DNA. The OMVs were then ultrasonically cleaved and treated with DNase to remove DNA from the cavity. *G. parasuis*, OMVs and OMVs without DNA were then used to infect RAW264.7 or 3D4/21 cells separately for 12 h. Compared with those in the OMV group, the mRNA transcription of IFN-β and the production of IFN-β in the OMVs without DNA group were significantly lower (Figures 3G, H and I). These results indicate that the DNA of *G. parasuis*, which is carried by OMVs, is the key component by which *G. parasuis* induces the production of type I interferon in macrophages.

### OMVs are transported in pulmonary alveolar macrophages (PAMs) mainly via dynamin-dependent endocytosis

**Figure 4:**
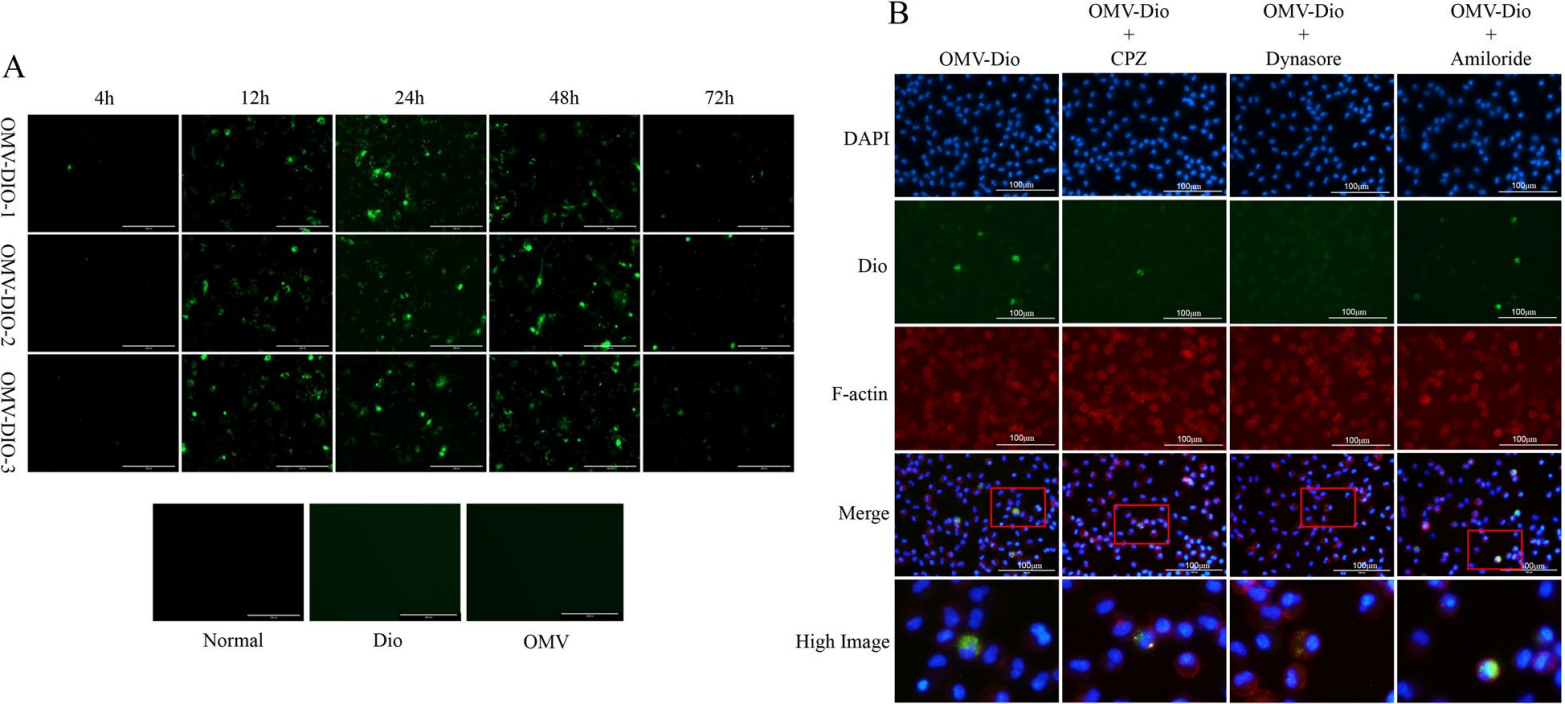
**OMVs are transported in PAMs mainly via dynamin-dependent endocytosis. A** The fluorescence levels of PAM cells treated with 50 μg/mL OMVs-Dio for 4, 12, 24, 48 or 72 h. The scale bar represents 50 μm. **B** *G. parasuis* OMVs (50 μg/mL) labeled with the lipophilic stain Dio (green) were added to the vascular endothelial cells for 24 h after the cells had been pretreated with the indicated endocytosis inhibitors. All the cells were fixed in paraformaldehyde and then dyed with DAPI (blue) and F-actin (red). Scale bars are 100 μm. The data are representative of three independent experiments with similar results (**A** and **B**).

Dio is 3′,3′-dioctadecyloxacarbonine perchlorate, also known as DiOC18(3), which is a lipophilic stain that is used to label *G. parasuis* OMVs. Figure 4A shows that *G. parasuis* OMVs-Dio were taken up by PAMs in a time-dependent manner. The cellular OMV-Dio fluorescence level increased with time, peaking at 24 h but rapidly weakening at 72 h. The uptake of *G. parasuis* OMVs was significantly reduced by dynasore, an inhibitor of dynamin [23]. Chlorpromazine (CPZ) is an inhibitor of clathrin-mediated endocytosis [24]. It could also reduce *G. parasuis* OMV uptake. However, amiloride, an inhibitor of micropinocytosis, did not reduce OMV uptake [25] (Figure 4B). These results demonstrated that *G. parasuis* OMVs are internalized by PAMs, partially via clathrin-mediated endocytosis and mainly via dynamin-dependent endocytosis, but not by micropinocytosis.

### OMVs of *G. parasuis* activate the cGAS–STING–IRF3 pathway

**Figure 5:**
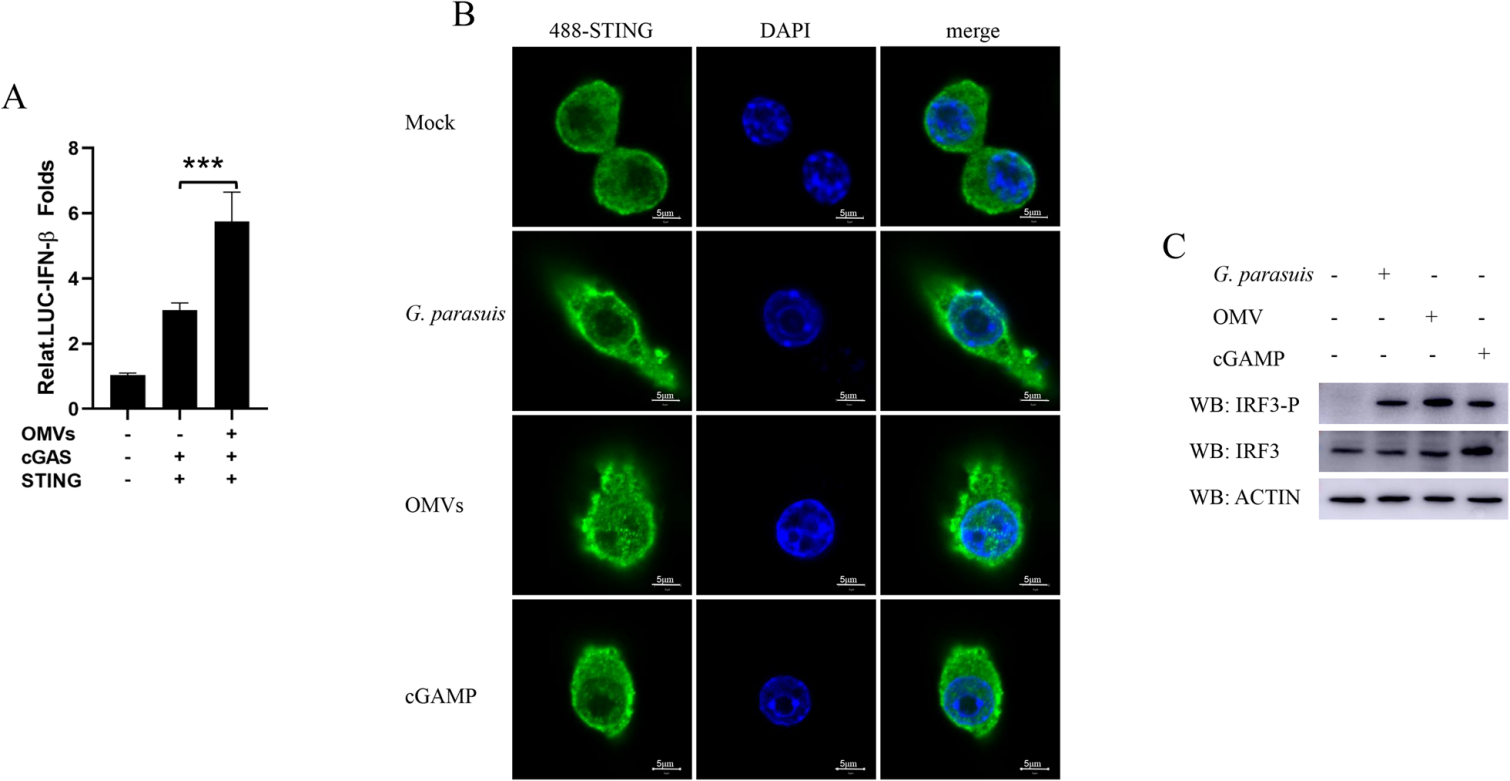
**OMVs of** ***G. parasuis*** **activate the cGAS–STING–IRF3 pathway. A** HEK293T cells were transfected with an IFN-β luciferase (Luc) reporter, an internal control, Renilla-TK Luc, and two plasmids individually expressing cGAS and STING, and the OMVs were added 24 h after transfection. The IFN-β promoter activities were measured after the cells were stimulated with OMVs for 12 h. **B** RAW264.7 cells were stimulated with either 10 MOI *G. parasuis*, 25 μg/mL OMVs or 10 μg/mL cGAMP for 12 h. The subcellular localization of STING (green) was observed via a confocal screen. Scale bars are 5 μm. **C** RAW264.7 cells were stimulated with either 10 MOI *G. parasuis*, 25 μg/mL OMVs or 10 μg/mL cGAMP for 12 h. The cells were subsequently collected, and the protein levels of IRF3-P, IRF3 and actin were detected via western blotting. ***p < 0.001, one-way ANOVA followed by the Bonferroni post-test (**A**). The data are expressed as the means ± SDs of triplicate assays (**A**) or are representative of three independent experiments with similar results (**B** and **C**).

cGAS is a versatile DNA receptor found in the cytoplasm, inner plasma membrane leaflet, and nucleus [26–28]. It plays a critical role in detecting various DNA forms within the cytoplasm, including those from DNA viruses, retroviruses, and bacteria, as well as the host's DNA, such as mitochondrial DNA or genomic DNA from deceased or damaged cells. This recognition activates a signaling cascade that results in the production of type I IFNs and inflammatory cytokines. To determine whether *G. parasuis* OMVs stimulate type I IFN production through the cGAS–STING pathway, luciferase reporter assays were conducted. HEK293T cells were transfected with an IFN-β luciferase (Luc) reporter, a Renilla-TK Luc internal control, or two plasmids expressing cGAS or STING. After 24 h of transfection, various doses of OMVs were introduced. The data presented in Figure 5A indicate that OMVs enhanced IFN-β promoter activity, which is driven by cGAS and STING, in a dose-dependent manner. Previous studies have demonstrated that upon activation, STING migrates from the endoplasmic reticulum to the nucleus, travels through the Golgi apparatus and accumulates near the nucleus. Hence, we separately stimulated RAW264.7 cells for 12 h with either 10 MOI *G. parasuis*, 25 μg/mL OMVs or 10 μg/mL cGAMP and observed the subcellular localization of STING via a confocal screen. As shown in Figure 5B, compared with those in the mock group, STING accumulated around the nucleus after *G. parasuis* and its OMVs were used to infect RAW264.7 cells for 12 h. The change in the subcellular localization of STING was consistent with the results of the cGAMP group. Once STING is activated, TANK-binding kinase 1 (TBK1) is recruited and phosphorylated at serine 365 [29–32]. Subsequently, activated TBK1 phosphorylates and dimerizes IRF3 and changes its sublocation from the cytoplasm to the nucleus to promote type I IFN production [33, 34]. Hence, we stimulated PAMS cells for 12 h with *G. parasuis*, OMVs or cGAMP separately. Western blotting was performed to detect the phosphorylation of IRF3. As shown in Figure 5C, *G. parasuis* and its OMVs activated the phosphorylation of IRF3 to generate cGAMP. These results indicate that the OMVs of *G. parasuis* activate the cGAS–STING–IRF3 pathway.

### Knocking down STING inhibits *G. parasuis*- and *G. parasuis*-induced IFNβ levels in macrophages

**Figure 6:**
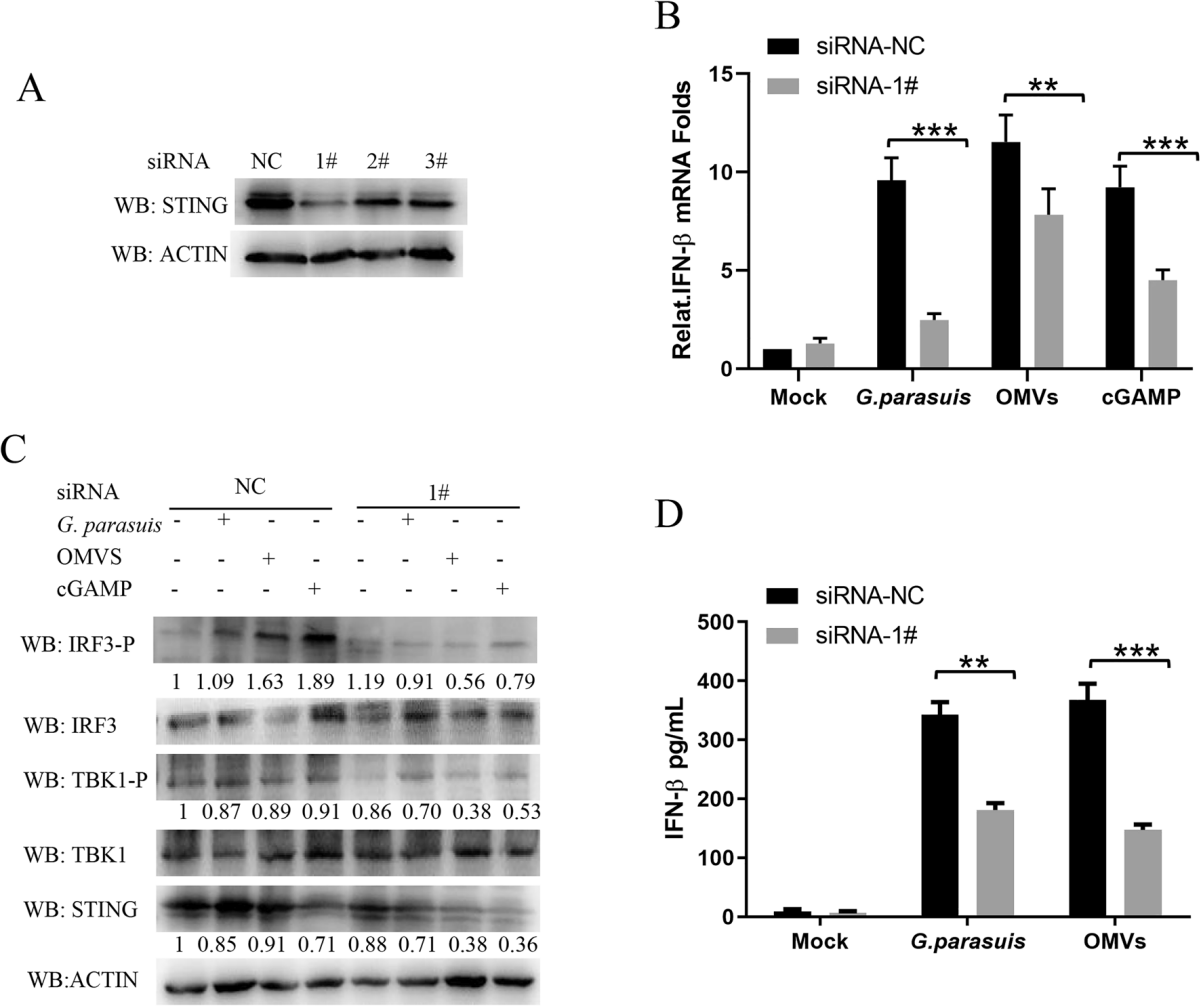
**Knocking down STING inhibits** ***G. parasuis-*** **and** ***G. parasuis*** **OMV-induced IFNβ expression in macrophages. A** RAW264.7 cells were transfected with three siRNAs (1#, 2#, and 3#) targeting the STING gene or a negative control (NC) siRNA for 24 h. The siRNA knockdown efficiency was analyzed via western blotting. **B** RAW264.7 cells were transfected with negative siRNA sequence (NC) or #1 siRNA for 24 h. Then, the cells were stimulated with either *G. parasuis* at an MOI of 10, 25 μg/mL OMVs or 10 μg/mL cGAMP for 12 h. The mRNA levels of IFN-β in the cells were detected by qPCR. **C** RAW264.7 cells were transfected with negative siRNA sequence (NC) or #1 siRNA for 24 h.Then, the cells were stimulated with either *G. parasuis* at an MOI of 10, 25 μg/mL OMVs or 10 μg/mL cGAMP for 12 h. The cells were collected, and the protein levels of IRF3-P, IRF3, TBK1, TBK1-P, STING and ACTIN were detected via western blotting. The gray degree values of IRF3-P, TBK1-P, STING and actin were analysed by Image J and homogenized the gray degree values of IRF3-P, TBK1-P and STING band according to ACTIN protein. **D** RAW264.7 cells were transfected with either the negative control (NC) sequence or #1 siRNA for 24 h. Then, the cells were stimulated with either 10 MOI *G. parasuis* or 25 μg/mL OMVs for 24 h. The IFN-β levels in the cell supernatants were analyzed via ELISA. **p < 0.01, ***p < 0.001; two-way ANOVA (**B** and **D**) followed by the Bonferroni post-test. The data are representative of three independent experiments with similar results (**A** and **C**) or are expressed as the means ± SDs of triplicate assays (**B** and **D**).

To further confirm the activating effects of *G. parasuis* and its OMVs on type I IFN signaling, we designed and synthesized three siRNA sequences that target the swine STING gene. The knockdown efficiency of STING in PAMs was detected via western blotting. The results in Figure 6A show that #1 (5′-GCUGCUGUCCUGCUACUUCUA-3′) was the most efficient siRNA sequence, so it was used in subsequent experiments. We separately transfected PAM cells with NC siRNA or #1 siRNA for 24 h and then stimulated the cells with *G. parasuis* (MOI = 1), OMVs (25 μg/mL) or cGAMPs (5 μg/mL) for 16 h. The results revealed that siRNA-mediated knockdown of STING significantly reduced the mRNA levels of IFN-β in all the stimulated groups (Figure 6B). To determine whether *G. parasuis* and its OMVs could induce type I IFN production via the cGAS–STING–IRF3 signaling pathway, the activation of key adaptor proteins of the cGAS–STING–IRF3 signaling pathway was detected via western blotting. As shown in Figure 6C, the knockdown of STING reduced the phosphorylation levels of IRF3 and TBK1, which were stimulated by *G. parasuis* and its OMVs. After STING was knocked down, the amounts of IFNs secreted by the PAMs induced by *G. parasuis* and its OMVs were measured via ELISA. The results were consistent with those in Figures 6B, C. These results indicate that *G. parasuis* and its OMVs induce type I IFN production via the cGAS–STING–IRF3 signaling pathway.

### IFN-β inhibits the adhesion and invasion of *G. parasuis* in macrophages

**Figure 7:**
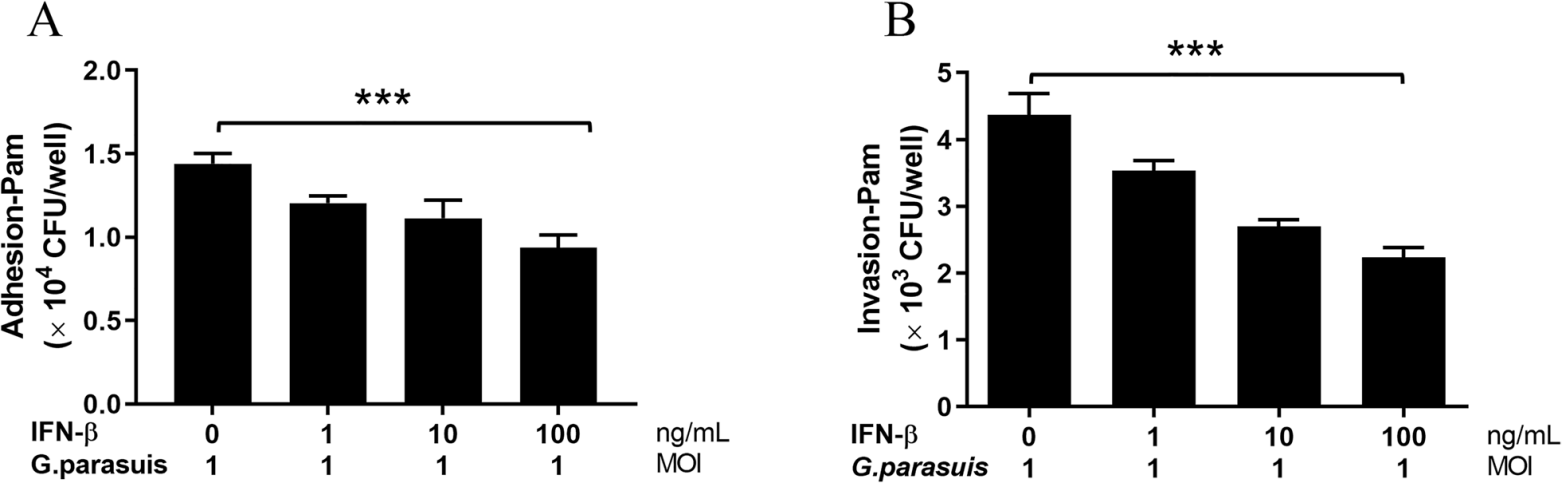
**IFNβ inhibits the adhesion and invasion of** ***G. parasuis*to macrophages. A** PAM cells were treated with 0, 1, 10 or 100 ng/mL swine IFN-β and then infected with *G. parasuis* at an MOI of 10 for 2 h. The adherence of *G. parasuis* s in each group was determined via plate counting. **B** PAMs were treated with 0, 1, 10 or 100 ng/mL swine IFN-β and then infected with *G. parasuis* at an MOI of 10 for 2 h. Extracellular *G. parasuis* were killed by adding 100 U/mL penicillin G and 100 μg/mL gentamicin for 1 h, and then each group of cells was washed five times with PBS and incubated for an additional 2 h. The invasion of *G. parasuis* in each group was determined via plate counting. *p < 0.05, **p < 0.01, ***p < 0.001; one-way ANOVA followed by the Bonferroni post-test (**A** and **B**). The data are expressed as the means ± SDs of triplicate assays.

The above results revealed that *G. parasuis* and its OMVs can induce type I IFN production in PAM cells. Type I IFNs are widely recognized for their ability to protect against viruses. However, the role of type I IFNs in the host defense against bacterial infections remains enigmatic [35]. To date, no reports have indicated a role for type I IFNs in *G. parasuis* infection. The bacterial adherence and invasion assays were performed as described for PAMs that had been treated with different doses of porcine IFN-β (0, 1, 10, or 100 ng/mL) and then infected with *G. parasuis* at an MOI of 10 for 2 and 4 h. As shown in Figure 7, the CFUs of *G. parasuis* significantly decreased depending on the dose of IFN-β, demonstrating that type I IFNs inhibit *G. parasuis* adhesion to and invasion of PAM cells.

## Discussion

Numerous bacterial products, such as lipopolysaccharides, DNA and RNA, can potently induce type I IFNs. Compared with studies on viruses, studies of bacterium-induced IFN production are still rare. Moreover, the contribution of type I IFNs to the host’s defense against bacterial infection remains unclear. *G. parasuis* is one of the important bacterial pathogens restricting the development of the global pig industry, and it has at least 15 serotypes [36]. Serovar 4 and serovar 5 strains were the dominant prevalent serotype of G. parasuis in the world [37]. In this study, we first found that *G. parasuis* H45 strain*,* which isolated from clinically infected pig and belongs to serovar 5, could induce type I IFN production in macrophages. As a new secretory system of Gram-negative bacterial vesicles, OMVs play an important role in the process of bacterial infection. In our previous studies, we confirmed that *G. parasuis* (H45 strain, serovar 5) can secrete OMVs during in vitro growth and that the diameter of OMVs ranges from 50 to 100 nm, so they are nanoparticles. Moreover, we found that OMVs induce strong inflammatory responses in macrophages. Moreover, IL-1β production, mRNA expression and secretion are stimulated by OMVs from *G. parasuis* in a dose- and time-dependent manner [6]. In this study, the OMVs induced IFN production in macrophages, similar to *G. parasuis* infection. These results suggest that OMVs play an important role in the interplay between *G. parasuis* and the host’s innate immune response.

Clearly, when intracellular bacteria invade and replicate in cells, they induce IFN production. However, since *G. parasuis* is an extracellular bacterium, how it induces IFN production needs more thorough investigation. OMVs constitute a novel independent secretory system of Gram-negative bacteria and are composed of lipopolysaccharides (LPS), phospholipids, peptidoglycans, outer membrane proteins, cell wall components, nucleic acids (DNA, RNA) and ionic metabolites [22]. LPS, DNA and RNA are the key PAMPs that are recognized by different PRRs to induce IFN production. Hence, we hypothesized that OMVs carrying these PAMPs could enter cells and induce interferon production. In this study, the lipophilic stain Dio was used to label *G. parasuis* OMVs. As shown in Figure 4, *G. parasuis* OMVs-Dio were internalized by PAMs partially via clathrin-mediated endocytosis but mainly via dynamin-dependent endocytosis in a time-dependent manner. Thus, the cellular uptake mechanism of *G. parasuis* OMVs is similar to that of *Escherichia coli* O157 and *Streptococcus suis* serotype 2 [38].

**Figure 8:**
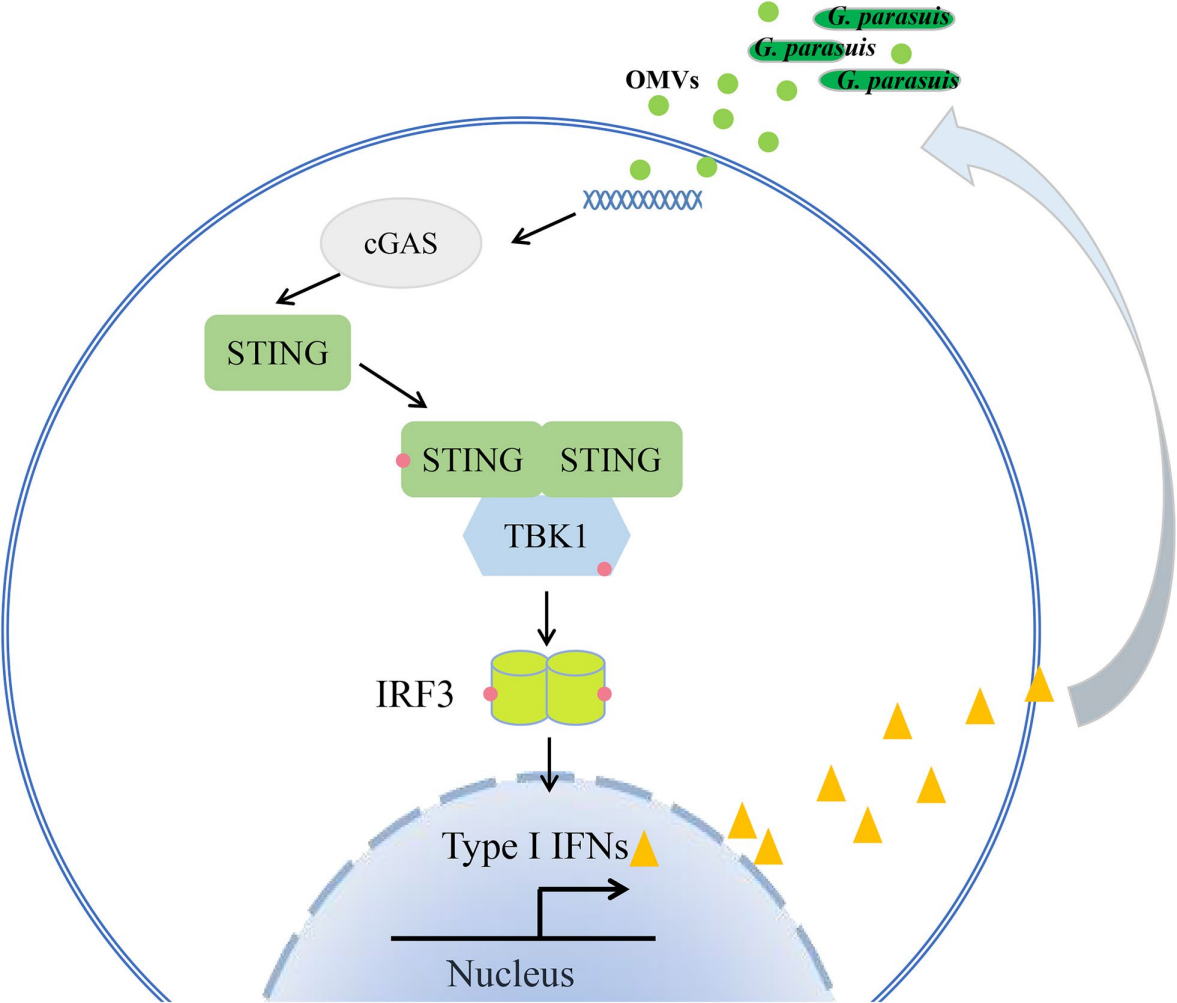
**Proposed pathway of type I IFN induction by outer membrane vesicles released from** ***G. parasuis*in macrophages.** The bacterium *G. parasuis* and its OMVs can induce type I interferon (IFN) production in macrophages. *G. parasuis* OMVs are internalized by PAMs partially via clathrin-mediated endocytosis and mainly via dynamin-dependent endocytosis. The internalized OMVs carry *G. parasuis* DNA, which induces IFN production in macrophages via the cGAS–STING–IRF3 pathway. Furthermore, type I IFNs inhibit *G. parasuis* adhesion to and invasion of macrophages.

After OMVs enter microphage cells, IFN production can be induced by either the LPS or the DNA that they carry. In this study, the OMVs were found to carry a large amount of *G. parasuis* nucleic acid. As shown in Figure 3B, 1 μg/mL OMVs contained approximately 135.2 ng of *G. parasuis* DNA, approximately 91% of which was wrapped in the vesicle cavity, whereas 9% of the DNA was located on the surface of the vesicle. After the DNA was removed, the level of IFN induction decreased, indicating that the DNA carried by OMVs was one of the main constituents that induced interferon production. In addition, although RNA was not detected in this study, the LPS or RNA carried on OMVs may also induce interferon production, so we will conduct more in-depth research on these two components in future studies. However, DNA is one of the main PAMPs that can be recognized by DNA sensors to induce the host’s innate immune response. cGAS is a novel nucleic acid transferase found in mammalian cells, and it is present in a wide variety of cells. cGAS recognizes bacterial DNA and catalyzes the synthesis of cGAMP, thereby activating STING and ultimately inducing the production of type I interferon [39]. Previous studies revealed that *Francisella, Listeria*, *Mycobacterium tuberculosis*, and *Chlamydia trachomatis* DNA depends on cGAS recognition to activate STING [1, 40–42]. In this study, we found that *G. parasuis* DNA carried by OMVs increased cGAS- and STING-induced IFN-β promoter activity in a dose-dependent manner. A confocal screen revealed that infection with *G. parasuis* light or OMVs could change the subcellular localization of STING from being scattered in the cytoplasm to being clustered around the nucleus (Figure 5). Moreover, the phosphorylation of IRF3 was detected via western blotting. To further confirm the activating effects of *G. parasuis* and its OMVs on type I IFN signaling via the cGAS–STING–IRF3 pathway, the STING gene was knocked down, and the production of type I IFN and the phosphorylation of TBK1 and IRF3 were determined. As expected, STING knockdown significantly reduced the mRNA levels of IFN-β, the amount of IFN secretion induced by *G. parasuis* and its OMVs in PAMs, and the phosphorylation levels of IRF3 and TBK1 (Figure 6). These results indicate that *G. parasuis* and its OMVs induce type I IFN production via the cGAS–STING–IRF3 signaling pathway. Since the OMV composition is complex, the cGAS–STING–IRF3 pathway may not be the only pathway involved in the induction of type I IFN production (Figure 8).

*Glaesserlla parasuis* is a common pathogenic bacterium in the upper respiratory tract of pigs, and it can only cause outbreaks when PRRSV, PCV, PRV or other viruses are present or when the body's resistance is reduced. The antiviral effects of interferon are well known. This study revealed that *G. parasuis* can enter cells via OMVs and induce interferon production. Infections by intracellular or extracellular bacteria, such as *Listeria monocytogenes*, *Francisella tularensis*, *Salmonella enterica* serovar Typhimurium, or *Staphylococcus aureus*, can be exacerbated by type I IFNs. In this study, we found that type I IFNs inhibited *G. parasuis* adhesion to and invasion of PAM cells via bacterial adherence and invasion assays. The results showed that IFN-β plays a protective role against *G. parasuis*. Studies of other bacteria have revealed that IFNs induce itaconic acid production to inhibit *Legionella pneumophila* infection [43]; IFN-β directly kills *Staphylococcus aureus* via its cationic and amphipathic properties on the molecular surface [44]; and during extracellular bacterial infections, such as *Helicobacter pylori* infection or polymymicrobial sepsis, type I IFNs promote Cxcl10-mediated cell recruitment to protect the host from infection [35]. However, the detailed mechanism by which type I IFN inhibits *G. parasuis* infection needs further study. This study is the first report on the effects of type I IFNs on *G. parasuis* infection. These results suggest that, as a resident bacterium in the respiratory tract, *G. parasuis* does not develop normally and that the OMVs produced by *G. parasuis* may induce type I IFNs and inhibit the proliferation of the bacteria. IFNs can be used for the prevention and control of *G. parasuis* infection, although the clinical effect of interferon in the prevention and treatment of *G. parasuis* infection still needs further research.

In this study, we first found that *G. parasuis* and its OMVs could induce IFN production in macrophages. Interestingly, the DNA of *G. parasuis*, which is carried by OMVs, is the key component of *G. parasuis* that induces macrophages to produce type I interferons by activating the cGAS–STING–IRF3 pathway. Furthermore, bacterial adherence and invasion assays revealed that type I IFNs inhibit *G. parasuis* adhesion to and invasion of PAM cells. The results of this study may contribute to our understanding of the role of OMVs in the infection and pathogenesis of *G. parasuis*.

## Data Availability

The original contributions presented in the study are included in the article. Further inquiries can be directed to the corresponding author.
